# On language production principles and the form of language: a más *cómo*, menos *por qué*

**DOI:** 10.3389/fpsyg.2013.00231

**Published:** 2013-04-30

**Authors:** Itziar Laka

**Affiliations:** Lingusitics and Basque Studies, Psycholingusitics Laboratory, University of the Basque Country (UPV/EHU)Vitoria-Gasteiz, Spain

The idea that the form of linguistic expressions is modulated by output requirements is widely accepted in current approaches in linguistics, including those taking “extremely nativist” positions. Thus, for instance, the Minimalist Program developed in recent years by Chomsky (1995) holds that language form is strongly determined by the requirements imposed on it by the two main interfaces it links: on the one hand the conceptual-intentional domain of meaning, and on the other, the sensorimotor domain by means of which linguistic expressions are externalized. Within psycholinguistics, consideration of the impact that processing requirements have on the form of sentences can be traced back to Yngve's *Depth Hypothesis* (Yngve, 1960), which identified an asymmetry in the incidence of left-branching and right-branching grammatical structures in English due to processing constraints. Thomas Bever (Bever, 1970), in a seminal paper generally acknowledged to pioneer modern psycholinguistics, put forth and discussed the hypothesis that the form of language reflects general cognitive laws, in such a way that mechanisms of language learning and processing partially determine the form of grammar. Fodor et al. (1974) integrated ideas in philosophy, psychology, and linguistics to explain what was called language performance, that is, language production and comprehension. At the time, the possibility that the form of grammatical structures might be determined by domain-general behavioral systems was thought of as an important challenge to linguistics. However, after decades of interdisciplinary language study, this view it is now endorsed to varying degrees by most linguists and cognitive scientists, including those in “classic theories” which also hold the view that certain central architectural aspects of human language are not dependent on experience but rather imposed on it by organism-internal biases (Berwick et al., 2013).

The question, then, is not whether output/input factors related to the production and perception (i.e., externalization) of language modulates linguistic form, but rather how they do it and whether that is all there is to linguistic form. Put differently, what needs to be determined is whether externalization factors can account for linguistic categories like Noun or Preposition that are distinct (though related to) conceptual entities; whether externalization conditions chisel discreteness into linguistic categories (phonemes, words, and phrases); whether externalization forces a combinatorial and hierarchical structure on language.

MacDonald (2013) presents us with a thorough discussion of some principles operating on language production, and suggests a significant impact of those principles on the form of language and grammar. The discussion is adequately framed within the general theme of the relative weight that organism internal factors and experience have in cognition. The paper opens with some reference to findings on motion perception and object-face recognition, and it summarizes the state of the art acknowledging that “While such accounts don't deny innate factors in perception, they are notable in ascribing a central role for experience in development and in adult performance.”

Two are the issues under discussion here. One is whether externalization (production) demands can *fully* account for language form, or whether externalization demands modulate language form in concurrence with other factors, some of which are organism internal and previous to experience, as in other aspects of cognition. If the first position were correct, then language would be truly distinct, and very much unlike motion perception, face recognition, and other cognitive functions in not involving innate, organism internal factors in its structure and development. The question is not whether experience plays a key role in development and adult performance, since no account of language denies that, but whether experience is all that is required to explain its nature, a more controversial view, particularly among scholars devoted to the study of language form.

The position defended by MacDonald does not appear to fall completely in this latter class, because the strong position at the start of the paper is softened into the claim that “the memory and planning demands of language production strongly affect the form of producers' utterances,” a statement that views externalization-production demands as one molding factor, leaving space for others. Indeed, few language researchers would feel uncomfortable agreeing with MacDonald “not that all aspects of language form and comprehension can be traced to the computational demands of language production, but rather that production's impact in these areas is so pervasive that understanding production becomes essential to explaining why language is the way it is, and why language comprehension works the way it does.” The interest of the current proposal lies in the details, that is, in showing *how* production can explain why language is the way it is. In scientific thinking, *a más cómo menos por qué*, the more we know about *how*, the less we need to ponder about *why* (Wagensberg, 2006).

MacDonald argues that language form is significantly molded by two general domain principles that seek to unburden computational requirements on the part of the speaker: *Easy First* and *Plan Reuse*. Easier elements are put first in the sequence, and speakers tend to use again the types of sentences they have just heard. In order to evaluate the predictive capabilities of these two principles, we need to know what types of elements are *easy*, and what a *plan* is. Regarding what is easy in language, it involves words that are easily retrieved, and since the reasons why a given word or phrase might be easy to retrieve can be many, the criteria for *easiness* are heterogeneous, including frequency, length (favoring shorter words and phrases), complexity (favoring syntactically less complex elements), importance for the speaker, previously mentioned material, newly mentioned material. MacDonald is well aware of the possible circularity and lack of predictive power of this principle; it is one thing to argue for analogies in the navigation or action-planning system regarding the easy-first principle, and another to show that it is operational in accounting for language form.

As examples, let us consider some aspects of linguistic form and how principles seeking to minimize difficulty during production might relate to them. We will consider a simple sentence form from English, shown in (1):

1. There are men in the room
  This is an existential sentence, a very simple type of statement, which nevertheless involves a number of interesting form-related particularities, as existential sentences do across languages. This is one reason why they have been extensively studied in linguistics (from Milsark, 1979; to Moro, 1997; among many others). From the perspective of easy-first, it would appear that both *there* and *men* could appear first in the sentence, because they are short and frequent words, with *men* having the advantage of being animate, and therefore conceptually very salient. But only *there* can take the first place in an existential English sentence. Consider how other attempts fail:
2.
  ^*^men are there in the room
  ^*^men are in the room there
  ^*^men there are in the room
  To account for these patterns, the *Plan Reuse* principle can be appealed to: English-speakers are used to hearing existential sentences in that form, and that is why they keep using them in such a form. The question that lingers is then why English should have chosen a *plan* that violates *easy-first* to begin with. Particularly, when it appears that nothing stands in the way of not saying that initial and apparently semantically redundant word *there*, as shown by the Spanish example in (3):
3.
  Hay hombres en la habitación
  Are men in the room
  “there are men in the room”
  One could also wonder about why, given the easy-first principle, this Spanish sentence doesn't turn into (4), placing the most salient piece of information at the start, particularly given the relative freedom or word order that Spanish displays in other regards:
^*^hombres hay en la habitación
  A purported example of *easy-first* in action, as discussed by MacDonald, is the choice between active and passive in English, which speakers can allegedly use to place easier elements early. However, these subtle choices, if they could account for the passive and active sentences produced in English, can hardly account for one central type of question that linguists try to answer, which revolves around the class of possible and impossible sentences, rather than preferred and dispreferred ones.
  Turning back to linguistic form, the central types of question linguists seek answers to involve contrasts like the one in (5) between Dutch and English. Why can Dutch create passive sentences out of intransitive (unergative) verbs as in (5a), whereas English or Spanish cannot (5b, c)?
5.
  - Er wordt door Jan getelefoneerd There was by Jan telephoned
  ^*^It/there was telephoned by John
  ^*^fue telefoneado por Juan
  Similarly, accounting for the form of language requires understanding how is it that passive constructions are characteristic of nominative languages like English, Spanish, and Dutch above, and how is it that they are missing in ergative languages, where instead of passives we find a different type of construction known as antipassive, illustrated in (6) for Inuit (examples from Bobaljik, 1992):
6.
  - Jaani-up tuktu tuqut-vaa Jaani-Erg caribou kill-3sg-3sg “Jaani killed a caribou”
  - Jaani tuktu-mik tuqut-si-vuq Jaani caribou-P kill-antp-3sg “Jaani killed (by/at) caribous”

In the last decades, linguistics has gained a deeper understanding of the central issues about the form of languages, and a large part of the explanation does not involve principles like *easy-first* or *plan reuse*. This, of course, does not mean that avoidance of computational burden is not operational in language production. But it does mean that a persuasive approach to linguistic form, whatever its type, must present coherent accounts of these types of typological correlations.

The times are ripe for a truly interdisciplinary quest to understand the complex nature of language, and there are many new outlooks that seek to find what the underlying forces are that make language so easy to use, but so hard to characterize (see Sanz et al., 2013 for a current forum). I thoroughly agree with MacDonald that the separation of psycholinguists who study production and perception mechanisms of language and linguists who study language form is a real and quite unfortunate one, but we both probably agree that this gulf has been bridged progressively in the last years, at least in some realms. This will undoubtedly increase the knowledge of the types of problems different language researchers seek to solve, and the often intricate details involved in the problems themselves.
